# Distinct Lipid A Moieties Contribute to Pathogen-Induced Site-Specific Vascular Inflammation

**DOI:** 10.1371/journal.ppat.1004215

**Published:** 2014-07-10

**Authors:** Connie Slocum, Stephen R. Coats, Ning Hua, Carolyn Kramer, George Papadopoulos, Ellen O. Weinberg, Cynthia V. Gudino, James A. Hamilton, Richard P. Darveau, Caroline A. Genco

**Affiliations:** 1 Department of Medicine, Section of Infectious Diseases, Boston University School of Medicine, Boston, Massachusetts, United States of America; 2 Department of Periodontics, School of Dentistry, University of Washington, Seattle, Washington, United States of America; 3 Department of Biophysics, Boston University School of Medicine, Boston, Massachusetts, United States of America; 4 Department of Microbiology, Boston University School of Medicine, Boston, Massachusetts, United States of America; The Ohio State University, United States of America

## Abstract

Several successful pathogens have evolved mechanisms to evade host defense, resulting in the establishment of persistent and chronic infections. One such pathogen, *Porphyromonas gingivalis*, induces chronic low-grade inflammation associated with local inflammatory bone loss and systemic inflammation manifested as atherosclerosis. *P. gingivalis* expresses an atypical lipopolysaccharide (LPS) structure containing heterogeneous lipid A species, that exhibit Toll-like receptor-4 (TLR4) agonist or antagonist activity, or are non-activating at TLR4. In this study, we utilized a series of *P. gingivalis* lipid A mutants to demonstrate that antagonistic lipid A structures enable the pathogen to evade TLR4-mediated bactericidal activity in macrophages resulting in systemic inflammation. Production of antagonistic lipid A was associated with the induction of low levels of TLR4-dependent proinflammatory mediators, failed activation of the inflammasome and increased bacterial survival in macrophages. Oral infection of ApoE^−/−^ mice with the *P. gingivalis* strain expressing antagonistic lipid A resulted in vascular inflammation, macrophage accumulation and atherosclerosis progression. In contrast, a *P. gingivalis* strain producing exclusively agonistic lipid A augmented levels of proinflammatory mediators and activated the inflammasome in a caspase-11-dependent manner, resulting in host cell lysis and decreased bacterial survival. ApoE^−/−^ mice infected with this strain exhibited diminished vascular inflammation, macrophage accumulation, and atherosclerosis progression. Notably, the ability of *P. gingivalis* to induce local inflammatory bone loss was independent of lipid A expression, indicative of distinct mechanisms for induction of local versus systemic inflammation by this pathogen. Collectively, our results point to a pivotal role for activation of the non-canonical inflammasome in *P. gingivalis* infection and demonstrate that *P. gingivalis* evades immune detection at TLR4 facilitating chronic inflammation in the vasculature. These studies support the emerging concept that pathogen-mediated chronic inflammatory disorders result from specific pathogen-mediated evasion strategies resulting in low-grade chronic inflammation.

## Author Summary

Several human pathogens express structurally divergent forms of lipid A, the endotoxic portion of lipopolysaccharide (LPS), as a strategy to evade host innate immune detection and establish persistent infection. Expression of modified lipid A species promotes pathogen evasion of host recognition by Toll-like receptor-4 (TLR4) and the non-canonical inflammasome. The Gram-negative oral anaerobe, *Porphyromonas gingivalis*, expresses lipid A structures that function as TLR4 agonists or antagonists, or are immunologically inert. It is currently unclear how modulation of *P. gingivalis* lipid A expression contributes to innate immune recognition, survival, and the ability of the pathogen to induce local and systemic inflammation. In this study, we demonstrate that *P. gingivalis* expression of antagonist lipid A species results in attenuated production of proinflammatory mediators and evasion of non-canonical inflammasome activation, facilitating bacterial survival in the macrophage. Infection of atherosclerosis-prone ApoE^−/−^ mice with this strain resulted in progression of chronic inflammation in the vasculature. Notably, the ability of *P. gingivalis* to induce local inflammatory bone loss was independent of lipid A modifications, supporting distinct mechanisms for induction of local versus systemic inflammation. Our work demonstrates that evasion of immune detection at TLR4 contributes to pathogen persistence and facilitates low-grade chronic inflammation.

## Introduction

Host recognition of Gram-negative bacteria occurs via detection of LPS expressed on the bacterial membrane by the innate immune receptor, TLR4 [1]. This initial recognition is critical for instructing host immunity and promoting an inflammatory response that eradicates the pathogen from the host [1], [2]. However, a number of Gram-negative organisms have evolved mechanisms to modify their lipid A species, the component of bacterial LPS that directly activates the TLR4 complex, as a strategy to evade immune detection and establish infection [3]. Lipid A is initially synthesized as a β-1′,6-linked disaccharide of glucosamine that is phosphorylated and fatty acylated [4]. An unmodified version of this lipid A structure is typically expressed by *E. coli* and induces a robust inflammatory response [5]. Modifications to this basic lipid A structure are observed in alterations to acyl chains or terminal phosphate groups [6]. *Helicobacter pylori* [7], *Legionella pneumophila* [8], *Yersinia pestis* [9], and *Francisella novicida* [10] express underacylated lipid A moieties, in comparison to the canonical LPS expressed by *E. coli*, and are poorly recognized by TLR4. *Yersinia pestis* [11] and *Francisella tularensis* [12] expression of structurally divergent forms of lipid A is highly regulated by local environmental conditions such as temperature, allowing these pathogens to adapt to harsh environmental conditions in the host. It has been postulated that the ability of these pathogens to cause persistent infection and severe disease is partially due to evasion of host immune detection at TLR4 [13].

Recently, it has been revealed that in addition to evasion of TLR4 signaling, lipid A modifications promote evasion of the non-canonical inflammasome by preventing activation of caspase-11 [14]. Activation of the inflammasome is characterized by the production of the proinflammatory mediators IL-1β and IL-18 and is associated with downstream events such as pyroptosis [15]. Due to its role in host innate defense, a number of pathogens have evolved strategies to evade activation of this complex [16]. Pathogen evasion of inflammasome activation has been proposed to serve a dual role: to dampen cytokine production and to prevent host cell death in order to provide an intracellular niche for the pathogen to survive [17]. One pathogen that has successfully adapted to evade the inflammasome is *H. pylori*, through expression of its tetra-acylated lipid A [14].

The oral pathogen *Porphyromonas gingivalis* weakly activates TLR4 through expression of a heterogeneous LPS that contains lipid A structures that vary in the number of phosphate groups and the amount and position of lipid A fatty acids [18], [19]. *P. gingivalis* expresses underacylated lipid A structures that can be penta-acylated forms, conferring TLR4 agonistic activity, or tetra-acylated forms, functioning as TLR4 antagonists, or are non-activating [20], [21]. These structures typically express mono- or di-phosphate terminal groups. Expression of divergent structural moieties by *P. gingivalis* changes depending on growth phase, temperature, and levels of hemin [22]–[24]. Recently, it has been demonstrated that *P. gingivalis* also expresses a unique non-phosphorylated, tetra-acylated lipid A that is regulated by levels of hemin [23]. During hemin-deplete conditions, *P. gingivalis* utilizes endogenous lipid A 1- and 4′-phosphatase activities to express a non-phosphorylated, tetra-acylated lipid A that is immunologically inert at the TLR4 complex, as well as a mono-phosphorylated, penta-acylated lipid A that functions as a weak TLR4 agonist [22], [23], [25]. Under hemin-replete conditions, the activity of 1-phosphatase is suppressed, resulting in the expression of a mono-phosphorylated, tetra-acylated lipid A species that functions as TLR4 antagonists [23]. Expression of these different structural types is believed to allow *P. gingivalis* to evade immune detection at TLR4 [26].

A hallmark of chronic infection with *P. gingivalis* is the induction of a local inflammatory response that results in destruction of supporting tissues of the teeth and resorption of alveolar bone [27]–[29]. In addition to inflammation induced at the initial site of infection, *P. gingivalis* has been associated with systemic diseases such as diabetes, pre-term birth, pancreatic cancer, and cardiovascular disease [30]–[32]. *P. gingivalis* has been detected in human atherosclerotic lesions and shown to be viable in atherosclerotic tissue [33]–[35]. Studies from our laboratory have validated human studies by demonstrating that oral infection of atherosclerosis-prone ApoE^−/−^ mice with *P. gingivalis* results in local oral bone loss and systemic inflammation in atherosclerotic lesions [36]. We have demonstrated that *P. gingivalis-*induced oral inflammatory bone loss and acceleration of systemic inflammation and atherosclerosis is dependent on TLR2 signaling [37], [38]. *P. gingivalis* engages TLR2 through the expression of several outer membrane components that include lipoprotein, major and minor fimbriae, and phosphorylated dihydroceramides [39]–[41]. The unique ability of *P. gingivalis* to induce TLR2 signaling and to evade TLR4 signaling has been proposed to enable this organism to cause low-grade persistent infection [42]; however, the expression of multiple *P. gingivalis* lipid A structures simultaneously has complicated the interpretation of how distinct lipid A moieties contribute to chronic inflammation [22].

To define the role of distinct lipid A species in *P. gingivalis* evasion of TLR4 signaling, innate immune recognition, survival, and the ability of the pathogen to induce local and systemic chronic inflammation, we constructed genetically modified strains of *P. gingivalis* that lack either 1- or 4′-phosphatase activity [23]. These resulting strains express lipid A species that are not under genetic regulation and function as TLR4 agonists or TLR4 antagonists. Utilizing these strains, we demonstrate that *P. gingivalis* expression of antagonist lipid A species results in attenuated production of proinflammatory mediators and evasion of non-canonical inflammasome activation, facilitating bacterial survival in the macrophage. Infection of atherosclerosis-prone ApoE^−/−^ mice with this strain resulted in progression of chronic inflammation in the vasculature. Notably, the ability of *P. gingivalis* to induce local inflammatory bone loss was independent of lipid A modifications, supporting distinct mechanisms for induction of local versus systemic inflammation. Collectively, these results indicate that expression of *P. gingivalis* lipid A structures that fail to engage TLR4 or function as TLR4 antagonists enables this pathogen to evade host innate immune detection and induce inflammation at sites distant from infection.

## Results

### *P. gingivalis* strain 381 modifies its lipid A structures through expression of endogenous lipid A 1- and 4′-phosphatase activities

**Figure 1:**
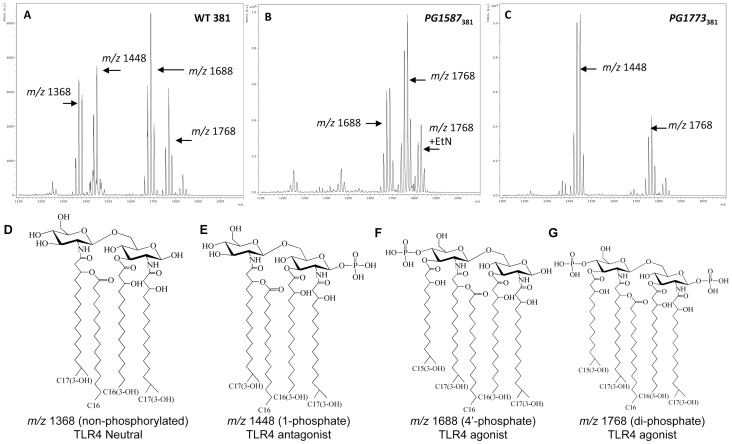
*P. gingivalis* strain 381 utilizes endogenous lipid A 1- and 4′- phosphatase activities to modify lipid A species and evade TLR4 activation. Lipid A isolated from *P. gingivalis* wild-type strain 381 (**A**) or lipid A mutant strains *PG1587*_381_ (**B**) and *PG1773*_381_ (**C**) were examined by MALDI-TOF MS. Arrows indicate the predominant lipid A species that are expressed for each strain (**A–C**). The major lipid A structures examined in this study have been identified in *P. gingivalis* as previously described (**D–G**) [19].

MALDI analysis of LPS isolated from *P. gingivalis* strain 381 revealed an ion cluster at m/z 1368 (**Figure 1A and 1D**). This structure represents the non-phosphorylated and tetra-acylated lipid A species that was predicted to be functionally inert at the TLR4 complex [23]. Additionally, we observed the expression of TLR4 antagonist (m/z 1448) and TLR4 agonist (m/z 1688 and m/z 1768) structures (**Figure 1A and 1E–G**). In order to examine the role of distinct lipid A species on the induction of inflammation, we constructed *P. gingivalis* strains lacking lipid A 1- and 4′- phosphatase activities in *P. gingivalis* 381. MALDI analysis of *P. gingivalis* strain *PG1587*_381_, that lacks 4′-phosphatase activity, revealed TLR4 agonist lipid A structures that centered at m/z 1768 and m/z 1688 (**Figure 1B and 1F–G**). MALDI analysis of *P. gingivalis* strain *PG1773*_381_, which lacks 1-phosphatase activity, revealed a TLR4 antagonist lipid A mass ion that was predominantly centered at ∼1448 m/z as well as the agonistic lipid A centered at ∼1768 m/z (**Figure 1C, 1E and 1G**).

**Figure 2:**
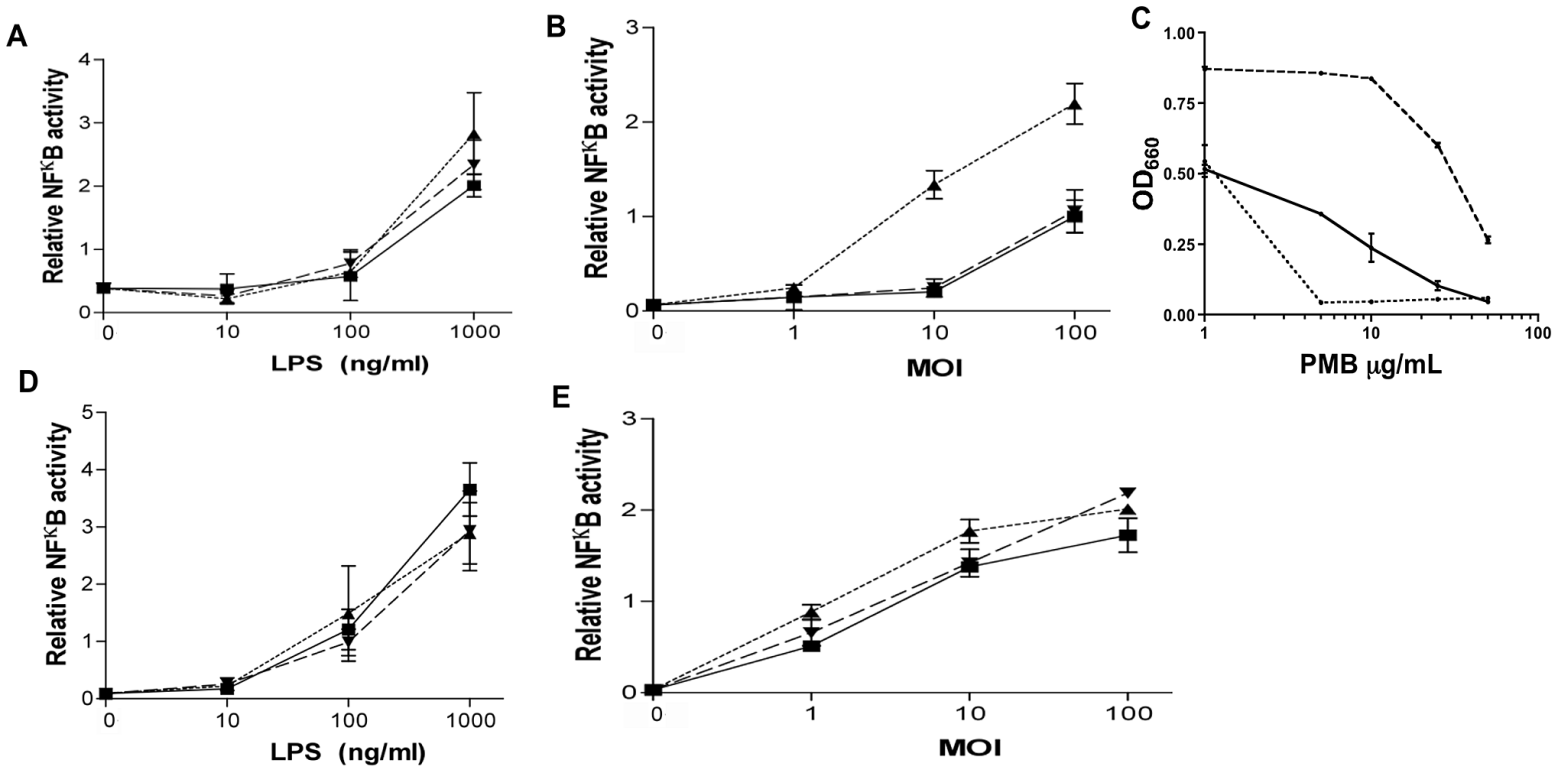
Lipid A phosphatase activity contributes to the ability of *P. gingivalis* to evade host innate immune defenses. HEK cells overexpressing mouse TLR4-MD2 (**A–B**) or TLR2 (**D–E**) were stimulated with corresponding LPS concentration purified from each strain (**A,D**) or whole bacterium (**B,E**) overnight. Relative NFkB activity indicates inducible firefly luciferase activity over the media control. Wild-type 381 and the lipid A mutants *PG1587*_381_ and *PG1773*_381_ were grown in BHI broth cultures overnight in the presence or absence of polymyxin B (PMB 1, 5, 10, 25, 50 µg/mL). Growth was measured by spectrophotometry (OD_660_) (**C**). Wild-type *P. gingivalis* (**solid**), *PG1587*_381_ (**dotted**), and *PG1773*_381_ (**dashed**). Graphs show the mean ± SEM of triplicate wells and are representative of two independent experiments. (See also **Figure S1**).

To confirm the predicted TLR4 activation phenotype of the lipid A expressed by wild-type 381 and the lipid A mutants, we stimulated HEK cells that overexpress mouse TLR4-MD2 with purified LPS from each strain. Notably, the LPS preparations purified from all three strains similarly activated mouse TLR4-MD2 (**Figure 2A**). In contrast, when live bacteria were used to stimulate the HEK cells only strain *PG1587*_381_ resulted in a significant increase in TLR4-dependent NF-κB activation as compared to wild-type 381 and *PG1773*_381_ (**Figure 2B**). These results suggest that the lipid A structures are differentially distributed within the bacterial cell membranes depending upon the strains, and that the relative localization of the specific agonistic and antagonistic lipid A forms to the outer cell membrane determines the respective abilities of the different strains to activate TLR4. The less potent lipid A forms (m/z 1368, 1448, 1688) may be primarily expressed on the bacterial outer membrane whereas the most potent lipid A form (m/z 1768) predominates in the inner membrane where it is initially synthesized prior to processing by phosphatases and deacylase(s).

In addition to direct impact on TLR4 activation, modifications in lipid A structure can significantly alter the ability of cationic peptides to kill bacteria. We have previously reported that two different strains of *P. gingivalis* (33277 and A7436) deficient in *PG1587* exhibit the most pronounced sensitivity to polymyxin B as compared to the wild-type and *PG1773* strains, consistent with a critical role of the lipid A 4′-phosphate in rendering bacteria susceptible to this drug [23], [43]. Assessment of these mutations in the 381 strains revealed a comparable pattern. Strain *PG1587*_381_ exhibits a pronounced susceptibility to polymyxin B while the *P. gingivalis* wild-type strain 381 and strain *PG1773*_381_ were relatively more resistant (**Figure 2C**). These data correlate well with the above TLR4 activation data indicating that lipid A structures localized in the outer membrane of the *PG1587*_381_ mutant contain 4′-phosphate (m/z 1688). In contrast, the *PG1773*_381_ strain is the most resistant, indicating the predominance of lipid A lacking 4′-phosphate in the outer membrane (m/z 1448). Wild-type 381 has an intermediate polymyxin B resistance phenotype consistent with an increased presence of lipid A containing 4′-phosphate as compared to strain *PG1773*_381_.

To verify that the mutant 381 strains exhibit phenotypes that are consistent with bacterial surface lipid A modifications rather than modifications of other surface virulence factors, we further assessed the ability of the *P. gingivalis* wild-type strain and the lipid A mutants to activate TLR2. All three *P. gingivalis* strains induced a similarly significant increase in NF-κB activation in HEK293 cells over expressing TLR2; however, we observed a slight decrease in the ability of the *P. gingivalis* wild-type strain 381 to activate TLR2 at lower MOIs (**Figure 2E** **and data not shown**). Stimulation of HEK-TLR2 cells with purified LPS isolated from the *P. gingivalis* wild-type strain 381 and the lipid A mutants resulted in equivalent activation of TLR2 (**Figure 2D**). These results were expected since *P. gingivalis* strongly activates TLR2 via expression of fimbriae and lipoproteins [39], [40]. To confirm that modification of lipid A structures in *P. gingivalis* strains *PG1587*_381_ and *PG1773*_381_ did not alter the expression of other outer membrane components, we examined the major fimbriae protein and activity of the cell-associated cysteine proteases, gingipain R and gingipain K. Similar levels of fimbriae expression were observed in *P. gingivalis* strains 381, *PG1587*_381_ and *PG1773*_381_ (**Figure S1-A-B**). We observed a slight decrease in gingipain activity (KGP and RGP) in *P. gingivalis* strain *PG1587*_381_ as compared to that observed in the wild-type strain (**Figure S1-C**). We did not observe significant differences in the growth of *P. gingivalis* strains *PG1587*_381_ and *PG1773*_381_ as compared to the wild-type strain (**Figure S1-D and data not shown**). Taken together, these results indicate that deletion of *PG1587* or *PG1773* alters the ability of the whole bacteria to activate TLR4 but does not alter the expression of other outer membrane components or the ability of the pathogen to activate TLR2. Therefore, the use of strains *PG1587*_381_ and *PG1773*_381_ in this study allowed us to assess the immunological consequences of differential lipid A expression by *P. gingivalis*.

### *P. gingivalis* expression of antagonistic lipid A attenuates induction of TLR4-dependent inflammatory mediators

**Figure 3:**
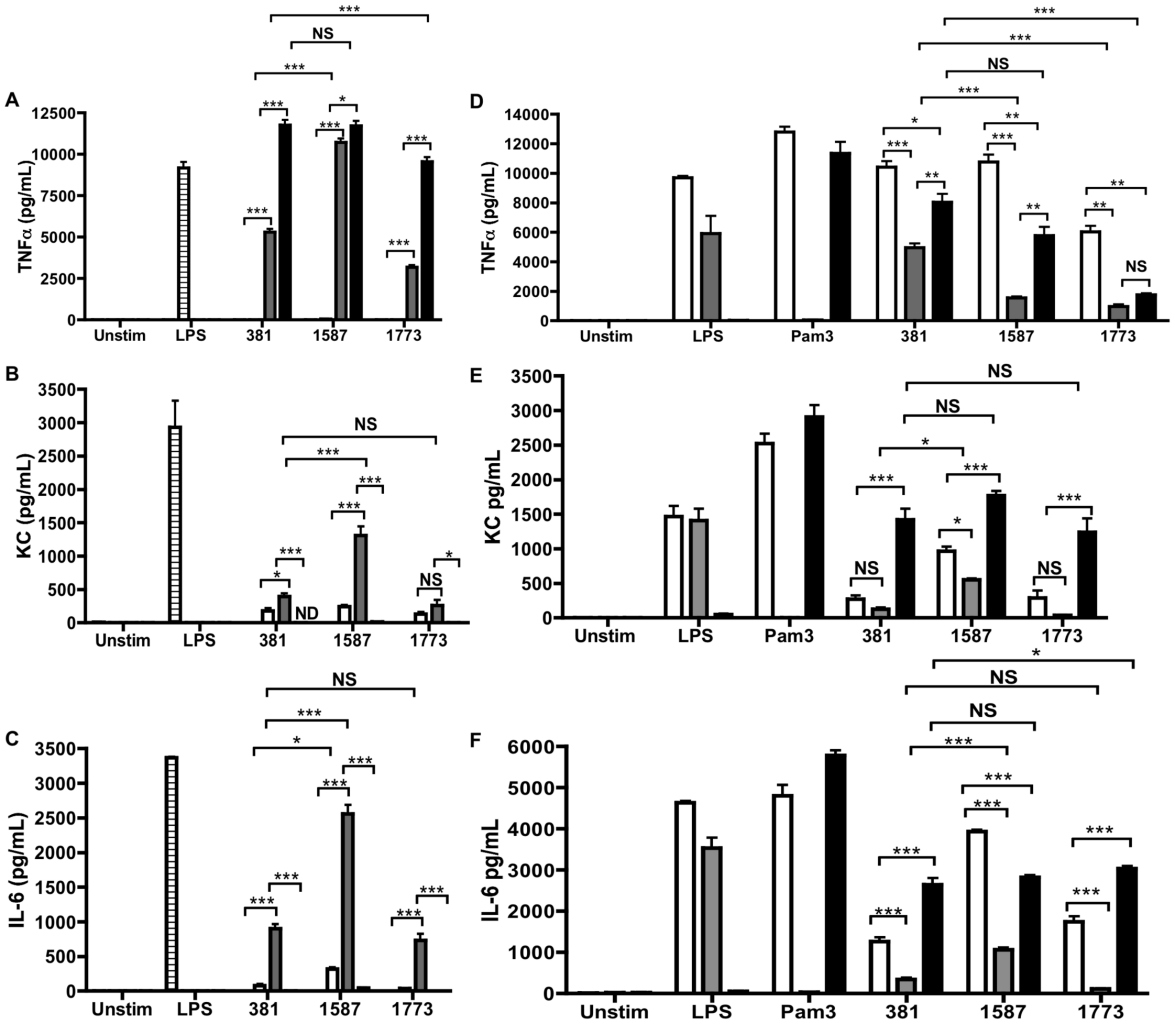
Expression of antagonistic or inert lipid A attenuates the production of NFkB-dependent proinflammatory mediators. BMDMs from C57BL/6 were stimulated with *P. gingivalis* wild-type strain 381 (381) or the lipid A mutant strains *PG1587*_381_ (1587) and *PG1773*_381_ (1773) at an MOI of 1 (**white**) 10 (**gray**) and 100 (**black**). Levels of TNFα (**A**), KC (**B**), and IL-6 (**C**) were assayed by ELISA at 24 h. BMDMs from wild-type C57BL/6 mice (**white**), TLR2-deficient (**gray**), or TLR4-deficient (**black**) were stimulated with *P. gingivalis* wild-type strain 381 or the lipid A mutant strains at an MOI of 100 (**D**) or MOI of 10 (**E–F**) and levels of TNFα (**D**) KC (**E**) and IL-6 (**F**) were assessed by ELISA at 24 h (See also **Figure S2**). Cells treated with *E. coli* LPS (100 ng/mL) or Pam3CysSk4 (1 µg/mL) for 5 h served as a positive control. Graphs show the mean ± SEM of triplicate wells and are representative of three independent experiments. *p<.05 **p≤.001 ***p<.0001; ANOVA with Bonferroni's posttest.

**Figure 4:**
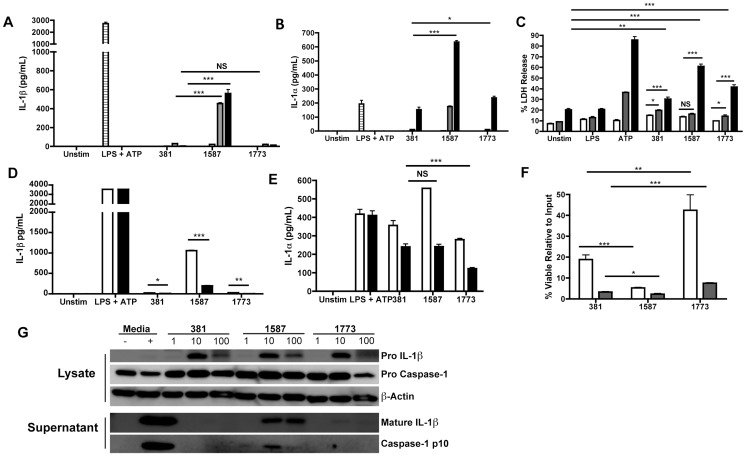
*P. gingivalis* evades activation of the inflammasome through expression of modified lipid A species. C57BL/6 WT BMDM were stimulated with *P. gingivalis* wild-type strain 381 or the lipid A mutant strains *PG1587*_381_ and *PG1773*_381_ at an MOI of 1 (**white**), 10 (**gray**) or 100 (**black**). Levels of IL-1β (**A**) and IL-1α (**B**) were assayed by ELISA at 24 h. LDH release (Promega) was assayed in BMDM stimulated with *P. gingivalis* wild-type strain 381 or the lipid A mutants at an MOI 100 at 2 h (**white**), 6 h (**gray**) and 24 h (**black**) (**C**). BMDM from wild-type (**white bars**) and caspase-11-deficient (**black bars**) mice were stimulated with *P. gingivalis* wild-type strain 381 or the lipid A mutants at an MOI of 100 and levels of IL-1β (**D**) and IL-1α (**E**) were assessed by ELISA at 24 h. Viable counts (CFU) of internalized *P. gingivalis* were determined by plating serial dilutions of macrophage lysates on blood agar plates at 2 h (**white**) and 6 h (**gray**) (**F**). Western blot analysis was performed on BMDM cell lysates to assess levels of the proform of IL-1β, pro Caspase-1 and β-actin at 24 h (**G**). Levels of mature IL-1β and active caspase-1 (p10) were detected in cell supernatants. BMDMs treated with media alone (−) or with *E. coli* LPS (100 ng/mL) for 5 h and then ATP (5 mM) for 20 m (+) served as the negative and positive control respectively. Graphs depict the mean ± SEM of triplicate wells and are representative of at least three independent experiments. * p<.05 **p≤.001 ***p<.0001; two-tailed unpaired t-tests. (See also **Figure S2**).

We examined the ability of the *P. gingivalis* strains lacking lipid A 1- and 4′-phosphatase activities to induce NF-κB-dependent proinflammatory cytokines in bone marrow-derived macrophages (BMDM). Stimulation of BMDM with *P. gingivalis* strain *PG1587*_381_ resulted in increased production of KC, IL-6, IL-1β, and IL-1α compared to *P. gingivalis* strains 381 and *PG1773*_381_ in a dose-dependent manner (**Figure 3B–C** **;** **Figure 4A–B**). In contrast, all three *P. gingivalis* strains induced significant levels of TNF-α. (**Figure 3A**). The role of TLR2 and TLR4 in the production of these inflammatory cytokines was assessed in BMDM obtained from TLR2- and TLR4-deficient mice. *P. gingivalis*-induced TNF-α production required both TLR2 and TLR4 signaling; however, TLR2 signaling was more dominant (**Figure 3D**). Additionally, we observed that the ability of *P. gingivalis* to induce IL-1β and IL-1α was dependent on both TLR2 and TLR4 signaling (**Figure S2**). In contrast, *P. gingivalis*-induced expression of KC and IL-6 were primarily dependent on TLR4 signaling (**Figure 3E–F**). We observed that *PG1587*_381_ induced enhanced KC and IL-6 levels in TLR2-deficient macrophages as compared to the *P. gingivalis* wild-type strain 381, suggesting the increased cytokine production we observed in wild-type BMDM was mediated via TLR4 signaling. Furthermore, we observed comparable KC and IL-6 levels in BMDM deficient in TLR4 following stimulation with all 3 strains of *P. gingivalis*, suggesting additional signaling through TLR2 in the absence of TLR4 or an inability of lipid A to antagonize production of these cytokines. Overall, these results indicate that expression of antagonistic or inert lipid A attenuates the production of NFκB-dependent proinflammatory mediators in macrophages.

### *P. gingivalis* evades inflammasome activation through expression of lipid A structures that evade TLR4 detection

IL-1β is considered an “alarm” cytokine and has been shown to be critical for host defense against infection [16], [44]. Previous studies have documented that *P. gingivalis* fails to induce significant levels of IL-1β production in macrophages [45], [46]. Thus, the increased production of IL-1β observed in macrophages stimulated with *P. gingivalis* strain *PG1587*_381_ was an unexpected finding (**Figure 4A**). IL-1β is first produced as an inactive zymogen, through activation of TLR signaling [47]. We observed that all three *P. gingivalis* strains induced expression of the proform of IL-1β (**Figure 4G**), which was dependent on TLR2 and TLR4 (**data not shown**). These results indicated that the attenuated production of IL-1β observed with *P. gingivalis* strains 381 and *PG1773*_381_ was not due to an obstruction of TLR-mediated synthesis of pro-IL-1β.

These findings led us to investigate the role of *P. gingivalis* lipid A modifications on inflammasome activation, the second signal required for production of mature and active IL-1β. Stimulation of macrophages with *P. gingivalis* strain *PG1587*_381_ resulted in activation of the inflammasome, as assessed by detection of the active caspase-1 p10 subunit in cell supernatants by Western blot analysis (**Figure 4G**). We also observed an increase in macrophage cell lysis following stimulation with *P. gingivalis* strain *PG1587*_381_ (**Figure 4C**), an event that is downstream from inflammasome activation. These results correlated with an inability of *PG1587*_381_ to survive in macrophages. We observed a significant decrease in survival of *P. gingivalis* strain *PG1587*_381_ in macrophages compared to *P. gingivalis* strains 381 and strain *PG1773*_381_ (**Figure 4F**).

Caspase-11 has emerged as an important mediator of inflammasome activation in Gram-negative bacterial infections [48], [49]. We thus examined the role of caspase-11 in *P. gingivalis*-induced IL-1β production. Interestingly, IL-1β production was completely ablated in macrophages deficient in caspase-11 following stimulation with *P. gingivalis* strain *PG1587*_381_ (**Figure 4D**), while TNFα levels were not altered (data not shown). In agreement with the requirement for caspase-11 in IL-1α production [50], we observed decreased production of IL-1α following stimulation of BMDM obtained from caspase-11-deficient mice with *P. gingivalis* strain *PG1587*_381_. However, we did not observe significant differences in the levels of IL-1α in BMDM obtained from caspase-11-deficient mice when stimulated with wild-type 381 compared to strain *PG1587*_381_ (**Figure 4E**). These results suggest distinct mechanism(s) for IL-1α production independent of lipid A modifications. Collectively, these findings indicate that expression of modified lipid A species by *P. gingivalis* facilitates evasion of non-canonical inflammasome activation through a caspase-11-mediated pathway and obstructs downstream events associated with activation of this complex.

### Alternative lipid A structures produced by *P. gingivalis* differentially influence systemic inflammation and local oral inflammation

To determine if the expression of modified lipid A by *P. gingivalis* is associated with the ability of the organism to promote chronic inflammation *in vivo*, we utilized a mouse model that mimics chronic *P. gingivalis* exposure as seen during human infection [51], [52]. Atherosclerosis-prone ApoE^−/−^ mice were orally infected with *P. gingivalis* strains 381, *PG1587*_381_, and *PG1773*_381_ and chronic inflammation at local (oral bone loss) and systemic (atherosclerosis) sites was evaluated [36].

**Figure 5:**
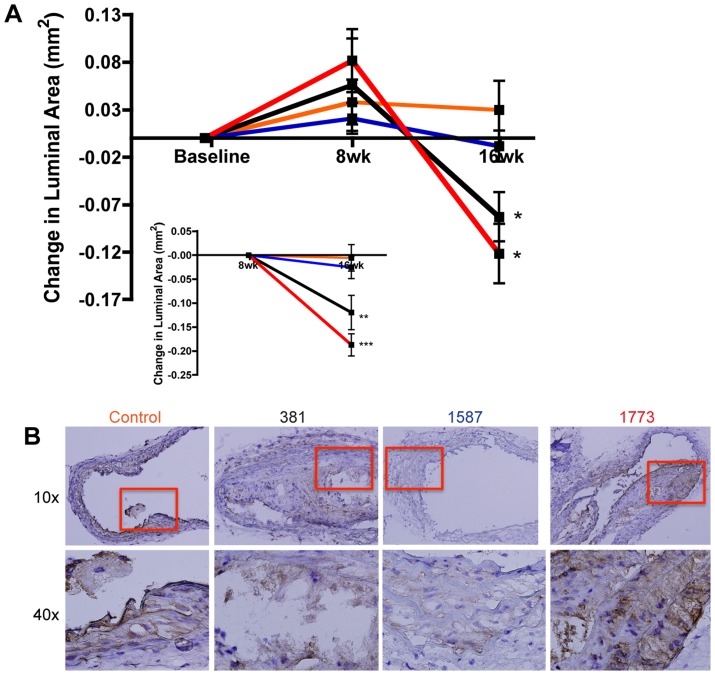
Expression of immunologically silent or antagonistic lipid A structures exacerbates atherosclerotic plaque progression in the innominate artery. Innominate arteries of ApoE^−/−^ mice were imaged by MRA at baseline (wk 0) and at 8 and 16 wks after first oral infection. The temporal change in luminal area (mm^2^) was calculated for individual mice normalized to baseline luminal area (n = 10–12/group) (**A**). Sham-infected ApoE^−/−^ (**orange**); 381-infected ApoE^−/−^ (**black**); *PG1587*_381_-infected ApoE^−/−^ (**blue**); *PG1773*_381_- infected ApoE^−/−^ (**red**). *Inset* - the temporal change in luminal area calculated for individual mice normalized to 8 wk luminal area (n = 10–12/group). *p≤.01 **p<.001 ***p<.0001; two-tailed unpaired t-tests compared to sham-infected and *PG1587*_381_-infected mice. (**B**) Representative images of the innominate artery with F4/80 staining (macrophages stain brown) and hematoxylin counterstaining for each group at 10× and 40× (n = 3/group) (See also **Figure S3**).

Plaque accumulation in the innominate artery was examined by magnetic resonance angiogram (MRA) throughout the course of the study to assess progression of site-specific inflammatory atherosclerosis. The innominate artery exhibits a high degree of lesion progression and expresses features of human disease including vessel narrowing and perivascular inflammation [53]. MRA at 8 weeks after oral infection resulted in an increase in the change of luminal area for mice infected with all 3 strains, indicating that the mice were still growing at this age (14–16 weeks of age) (**Figure 5A**). At 16 weeks after oral infection, MRA revealed that oral infection with *P. gingivalis* strains 381 and strain *PG1773*_381_ resulted in significant luminal narrowing compared to sham-infected controls (**Figure 5A**). In contrast, oral infection with *P. gingivalis* strain *PG1587*_381_ induced minimal luminal narrowing compared to sham-infected controls (**Figure 5A**). Furthermore, *P. gingivalis* strains 381 and *PG1773*_381_ induced progressive luminal narrowing from 8 to 16wks compared to strain *PG1587*_381_ and sham-infected controls (**Figure 5A** **** ***inset***).

Luminal narrowing was further validated by histological assessment of the innominate artery in postmortem sections. Hematoxylin and eosin staining of the innominate artery corresponding to the region of MRA analysis revealed an occlusion of the lumen in ApoE^−/−^ mice infected with *P. gingivalis* strain 381 (**Figure S3**), in agreement with our previous studies [53]. Oral infection with *P. gingivalis* strain *PG1773*_381_ resulted in plaque accumulation that was comparable to that observed with *P. gingivalis* strain 381 (**Figure S3**). Oral infection with *P. gingivalis* strain *PG1587*_381_ resulted in thickening of the vessel wall without occlusion of the vasculature (**Figure S3**). To assess macrophage infiltrate of the atherosclerotic lesions, histological sections of the innominate artery were stained with F4/80. We found oral infection with *P. gingivalis* strains 381 and *PG1773*_381_ resulted in an increase in macrophage accumulation in the innominate lesions compared to that observed with *P. gingivalis* strain *PG1587*_381_ and sham-infected controls (**Figure 5B**).

**Figure 6:**
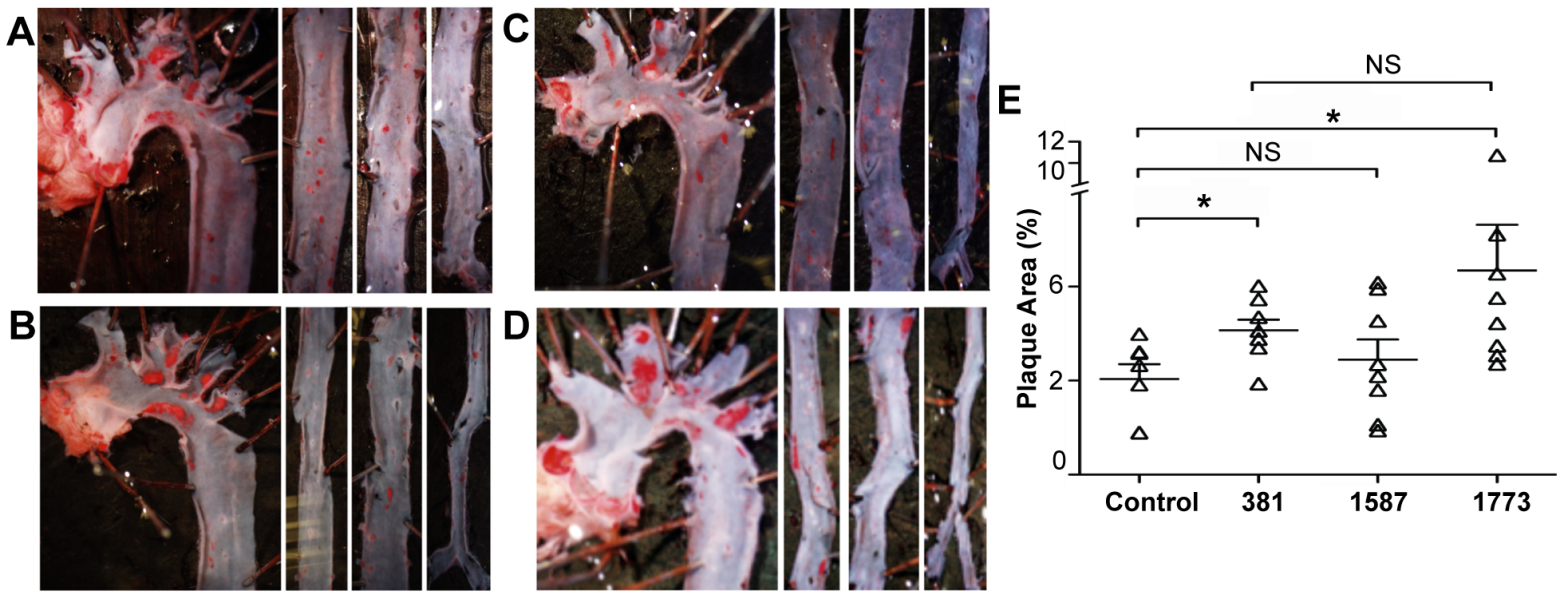
Expression of immunologically silent or antagonistic lipid A structures exacerbates atherosclerotic plaque progression in the aorta. Plaque area was determined from Oil Red O staining for lipids in *en face* aortic lesions 16 wk after first infection from (**A**) sham-infected, (**B**) wild-type 381, (**C**) *PG1587*_381_, and (**D**) *PG1773*_381_. (**E**) Quantification of lipid content within total aorta was calculated using ImageJ software (NIH) (n = 8/group). * p<.05; two-tailed unpaired t-tests. (See also **Figure S4**).

Lipid staining of *en face* aortas revealed that oral infection with *P. gingivalis* strains 381 and *PG1773*_381_ accelerated plaque accumulation compared to that observed in sham-infected mice (**Figure 6**). In contrast, oral infection with *P. gingivalis* strain *PG1587*_381_ induced minimal plaque accumulation (**Figure 6C**). The inability of *P. gingivalis* strain *PG1587*_381_ to elicit inflammatory disease pathology was not due to failed activation of immunity, since we observed an induction of the humoral response by *P. gingivalis* strain *PG1587*_381_ that was comparable to that induced by *P. gingivalis* strains 381 and strain *PG1773*_381_, as observed by serum levels of IgG1, IgG2b, IgG2c and IgG3 (**Figure S4**).

**Figure 7:**
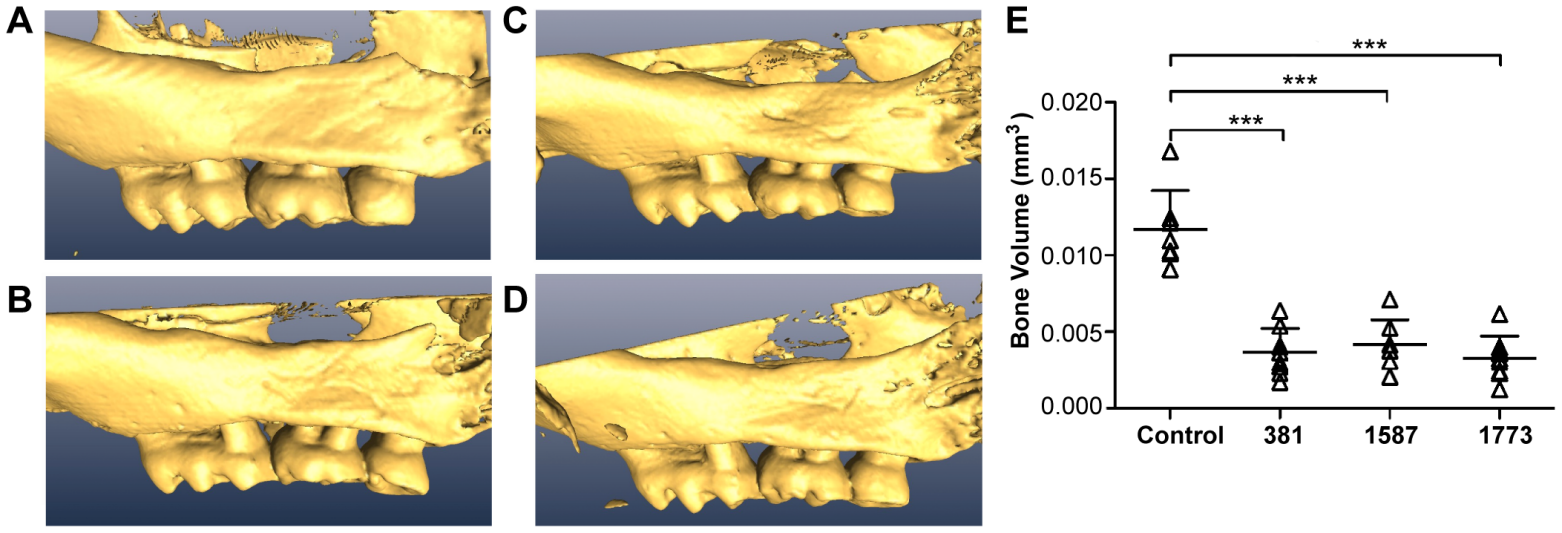
Expression of immunologically silent, agonistic or antagonistic lipid A does not alter the ability of the pathogen to induce oral bone loss. Maxillae were dissected (n = 8/group) 16 wk post initial infection and scanned on a micro CT 40 apparatus. Using AMIRA software, three-dimensional images were generated from micro CT scans. Representative images from (**A**) sham-infected mouse indicating no bone loss, (**B**) *P. gingivalis* wild-type strain 381-induced bone loss, (**C**) *PG1587*_381_, and (**D**) *PG1773*_381_. (**E**) Quantification of bone loss. ***p<.001; two-tailed unpaired t-tests.

Assessment of alveolar bone loss in infected mice revealed that *P. gingivalis* strains 381, *PG1587*_381_, and *PG1773*_381_ all induced oral bone loss at similar levels (**Figure 7**). These results are consistent with studies demonstrating a predominant role for TLR2 signaling in *P. gingivalis*-induced oral inflammatory bone loss [54], [55]. Collectively, these results indicate that expression of *P. gingivalis* lipid A structures that fail to engage TLR4 or function as TLR4 antagonists enables this pathogen to evade host innate immune detection and contributes to inflammation at sites distant from infection.

### Discussion

**Figure 8:**
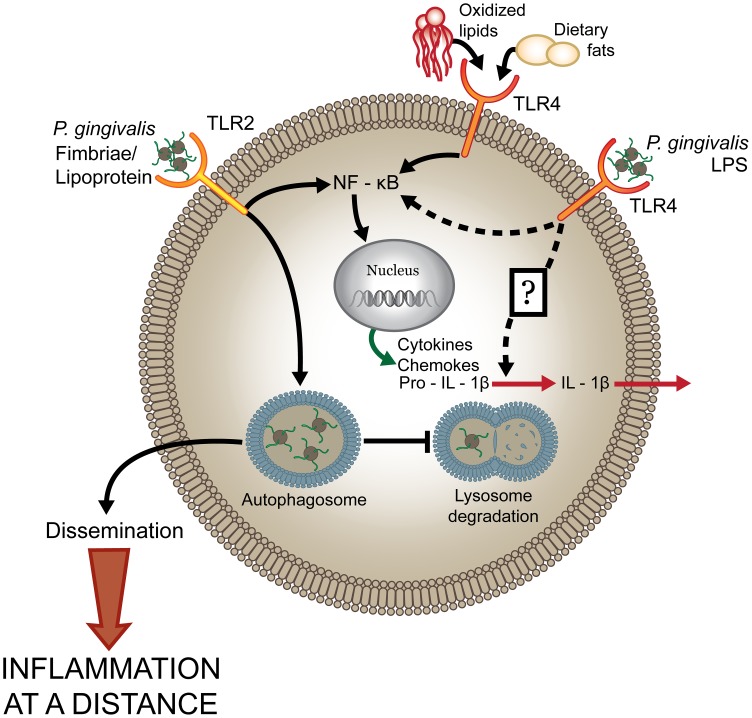
*P. gingivalis* dysregulates host cell immune activation facilitating systemic inflammation. The host predominantly senses *P. gingivalis* infection through engagement of TLR2 while the involvement of TLR4-dependent recognition is significantly impaired. Expression of antagonistic or immunologically inert lipid A by *P. gingivalis* attenuates production of proinflammatory mediators and prevents activation of the inflammasome (potentially through evasion of an unknown sensor) that facilitates intracellular survival. These events allow the pathogen to disseminate and to exacerbate systemic inflammation. In contrast, increased immunostimulatory potential at TLR4, through expression of an agonistic lipid A moiety, results in increased production of proinflammatory mediators, inflammasome activation, and reduced survival of the bacterium in macrophages leading to attenuated systemic inflammation.

In this study, we generated genetically defined strains of *P. gingivalis* expressing TLR4 agonist and antagonist lipid A species to examine the role of TLR4 evasion in *P. gingivalis*-induced chronic inflammation. Importantly, we determined that expression of TLR4 antagonist lipid A contributes to the ability of *P. gingivalis* to activate innate immunity and induce inflammation at systemic sites. Notably, induction of local inflammatory bone loss in response to *P. gingivalis* infection was not dependent on lipid A modifications, indicative of distinct mechanisms for the induction of local versus systemic vascular chronic inflammation. The expression of antagonistic or immunological inert lipid A species was associated with attenuated production of proinflammatory mediators and inflammasome activation, which correlated with increased bacterial survival in macrophages. We conclude that *P. gingivalis* evades TLR4-mediated bacterial clearance in the host, allowing it to exacerbate vascular inflammation (**Figure 8**).

We and others have reported that the expression of *P. gingivalis* lipid A is regulated by growth phase, temperature, and levels of hemin [22]–[24]. The expression of multiple lipid A structures has complicated the interpretation of the host response elicited by *P. gingivalis* LPS [22]. During growth under hemin-replete conditions, *P. gingivalis* expresses an antagonistic lipid A, due to the repression of 1-phosphatase activity [23]. The antagonist lipid A expressed by *P. gingivalis* has been demonstrated to antagonize *E. coli* LPS binding to TLR4 [56] and to dampen the cytokine response typically induced by other Gram-negative pathogens [45]. The use of *P. gingivalis* strain *PG1773*_381_ in this study allowed us to specifically define the role of the antagonistic lipid A (typically expressed under hemin-replete conditions) in the induction of chronic inflammation at both local and distant sites from infection. We observed that *P. gingivalis* strains 381 and *PG1773*_381_ induced comparable levels of inflammatory atherosclerosis suggesting that *in vivo P. gingivalis* is exposed to a hemin-replete environment and predominantly expresses an antagonistic lipid A that exacerbates chronic systemic inflammation.

Evasion of TLR4 signaling through lipid A modifications has been attributed to the ability of a number of highly pathogenic Gram-negative pathogens to cause disease [3]. For example, *F. novicida* expresses a tetra-acylated lipid A, with one phosphate group and does not induce a TLR4 response [10]. *Y. pestis* expresses a tetra-acylated lipid A that functions as a TLR4 antagonist at 37°C, which allows this pathogen to remain undetected in the bloodstream during early stages of infection [9], [11]. Generation of a strain of *Y. pestis* that expressed a more immunostimulatory LPS conferred a protective immune response against this pathogen [57]. Likewise, *Pseudomonas aeruginosa* expresses a modified LPS which promotes evasion of TLR4 signaling, favors intracellular survival, and has been postulated to contribute to chronic persistence in Cystic Fibrosis patients [58]. In agreement with these studies, we have shown that *P. gingivalis* modifies its lipid A structure in order to evade host defenses and establish chronic infection leading to persistent low-grade inflammation in the vasculature. Uniquely, *P. gingivalis* evasion of host innate immunity at TLR4 results in progression of inflammation at a site that is distant from local infection by gaining access to the vasculature.

A number of reports have proposed that *P. gingivalis* lipid A modifications are a mechanism for evasion of TLR4 signaling [6], [23], [26]. However, these studies utilized purified *P. gingivalis* LPS in an *in vitro* setting. To date, only this study and our recent study in a rabbit model of periodontitis [43] have begun to shed light on the immunological consequences of differential activation of the TLR4 complex by *P. gingivalis* LPS through the use of live bacteria that express a “locked” lipid A profile that is not responsive to growth conditions. Furthermore, we assessed the host response in the oral cavity and vasculature, physiologically relevant sites of chronic inflammation observed in humans. Notably, the use of purified LPS in our study resulted in discrepancies in TLR4 activation by isolated LPS versus the whole bacterium. These differences observed in the host response may be a reflection of LPS structure and composition on the cell surface, which leave us with new areas of investigation with regards to LPS translocation. Potentially, the less potent lipid A forms (m/z 1368, 1448, 1688) may be primarily expressed on the bacterial outer membrane and, consequently, render wild-type 381 and *PG1773*_381_ unable to activate TLR4. In contrast, strain *PG1587*_381_ is expected to exclusively accumulate lipid A TLR4 agonists (m/z 1688 and possibly m/z 1768) in the outer membrane since the presence of a lipid A 4′-phosphate precludes production of the lipid A antagonist (m/z 1448) or non-activating lipid A (m/z 1368) in this strain.

Overproduction of proinflammatory mediators and dysregulated inflammasome activation has been reported to contribute to inflammatory pathology in a number of chronic diseases [59]. Paradoxically, we observed that increased stimulation of TLR4 by *P. gingivalis* enhanced production of proinflammatory mediators and activation of the inflammasome, resulting in attenuated systemic inflammation (**Figure 8**). These results indicate that production of inflammatory mediators is protective against pathogen-mediated chronic inflammation, which is in agreement with our recent results that documented a protective role for TLR4 in *P. gingivalis-*mediated chronic inflammation at systemic sites [42]. This report, along with our current study, is in contrast to other studies elucidating the role of TLR4 in pathogen-mediated atherosclerosis progression. Infection of ApoE^−/−^ TLR4^−/−^ mice, fed a high-fat diet, with *C. pneumoniae* resulted in diminished atherosclerosis compared to ApoE^−/−^ infected mice [60]. Additionally, a previous study documented that common mechanisms of signaling via TLR2, TLR4 and MyD88 link stimulation by multiple pathogens and endogenous ligands to atherosclerosis progression [61], [62]. Therapeutic antagonism has been suggested to be beneficial in the treatment of chronic atherosclerosis [63]. However, we have demonstrated TLR4 antagonism exacerbates atherosclerosis progression. Collectively, our study highlights the complexity of chronic inflammatory pathways in diseases like atherosclerosis that are exacerbated by pathogen infection and further elucidate pathogen-specific mechanisms for chronic disease progression.

An important observation from this study was that *P. gingivalis* failed to activate the inflammasome. It has recently been reported that Gram-negative bacteria utilize a non-canonical pathway for inflammasome activation that is mediated by caspase-11 [48], [49]. Kayagaki et al. [14] have shown that *H. pylori*, whose tetra-acylated lipid A poorly activates the TLR4 complex, failed to trigger the non-canonical inflammasome. Although TLR4 signaling correlates with non-canonical inflammasome activity, this group reported that activation of the non-canonical inflammasome is independent of TLR4 signaling, further suggesting an unknown sensor in the cytosol that detects modified lipid A (**Figure 8**). We found that caspase-11 expression was essential for IL-1β production elicited by *P. gingivalis* strain *PG1587*_381_, pointing to a pivotal role for activation of the non-canonical inflammasome in *P. gingivalis* infection.

Pathogen evasion of inflammasome activation has been proposed to serve a dual role: to prevent IL-1β release and to circumvent host cell death in order to provide an intracellular niche for the pathogen to survive [17]. Indeed, we observed that the low levels of IL-1β induced by *P. gingivalis* strains 381 and *PG1773*_381_ correlated with an enhanced ability of these organisms to survive in macrophages. Likewise, the induction of relatively high levels of IL-1β by *P. gingivalis* strain *PG1587*_381_ correlated with decreased survival of this strain in macrophages. The enhanced survival we observed with wild-type 381 and *PG1773*_381_ are in agreement with our observation that both strains were relatively resistant to killing in the presence of the cationic antimicrobial peptide polymyxin B. In contrast, strain *PG1587*_381_ was unable to survive intracellularly and was rapidly killed. These results suggest that *P. gingivalis* utilizes multiple mechanisms concurrently to promote its adaptive fitness.

The ability of *P. gingivalis* to survive intracellularly in the macrophage is intriguing when considering the link between periodontal disease and systemic inflammatory conditions. We propose that *P. gingivalis* entry and survival into macrophages may be a mechanism for dissemination of the bacterium from the oral cavity to other systemic sites, such as the vasculature. We have recently identified *P. gingivalis* in blood myeloid dendritic cells of humans with chronic periodontitis, suggesting a role for blood myeloid dendritic cells in harboring and disseminating pathogens from the oral mucosa to atherosclerotic plaques [64]. The mechanism utilized by *P. gingivalis* for survival in myeloid cells remains elusive. Wang et al. [65] have shown that intracellular survival of *P. gingivalis* within macrophages is dependent upon TLR2-mediated entry into lipid rafts. Pathogens that hijack lipid rafts do not readily fuse with late endosomes and lysosomes and may potentially fuse to autophagosomes [66], [67]. Whether intracellular trafficking to autophagosomes is the mechanism for *P. gingivalis* intracellular survival and dissemination to distant sites remains unknown (**Figure 8**).

An interesting finding from this study was expression of *P. gingivalis* modified lipid A species did not alter the ability of the organism to induce oral inflammatory bone loss. These results are in agreement with previous studies that show that TLR4 is not needed for the induction of inflammatory bone loss, and it is predominantly mediated via TLR2 signaling [37], [68]. We also recently identified a TLR2- and TNF-dependent macrophage-specific mechanism for *P. gingivalis*-induced inflammatory bone loss *in vivo* [38]. In the current study, we observed comparable levels of TNFα were induced in macrophages stimulated with wild-type 381 and the lipid A mutants. In addition to the role of TNFα, it has been recently reported that *P. gingivalis* is able to induce oral bone loss at very low colonization levels, which triggers changes to the amount and composition of the oral commensal microbiota [69]. We have recently demonstrated in a rabbit model of periodontitis that lipid A phosphatases are required for both colonization of the rabbit and increases in the oral microbial load [43]. Whether this same mechanism for inflammatory bone loss is at play in our study remains to be determined.

It is well established that atherosclerosis progression is due to excessive production of proinflammatory mediators [70]. Although lipid deposition is considered a leading contributor to the inflammation, additional stimuli, such as infectious agents, have been considered as sources for the continuous inflammation [35]. In our study, we observed infection with *P. gingivalis* induced low levels of proinflammatory mediators but accelerated chronic inflammatory atherosclerosis. Thus, the question arises as to why a pathogen that induces low levels of inflammatory mediators would accelerate chronic inflammatory atherosclerosis. In a recent clinical trial for the inflammasome-mediated disease, Cryopyrin-associated periodic syndrome (CAPS), patients receiving the humanized monoclonal antibody, canakinumab, specific for IL-1β, had a 67% increased risk for infection compared to 25% of patients in the placebo group [71]. This clinical trial supports the results presented in our study that highlight a protective role for activation of innate immunity against low-grade chronic infection. An additional clinical trial was recently launched using canakinumab with the hypothesis that IL-1β inhibition will reduce major cardiovascular events in patients with preexisting coronary artery disease (CAD) [72]. Our results demonstrate that the potential benefit of long-term use of a neutralizing antibody to IL-1β in humans at high risk for atherosclerotic vascular disease must be substantial enough to counter the increased risk of infection [71]. Furthermore, future therapies need to be developed to consider the complexity of inflammatory pathways in chronic inflammation and the role of chronic infection in disease pathology.

## Materials and Methods

### Ethics statement

This study was carried out in strict accordance with the recommendations in the Guide for the Care and Use of Laboratory Animals of the National Institutes of Health. The protocol was approved by Boston University's Institutional Animal Care and Use Committee (IACUC) protocol numbers AN15312 and AN14348. Boston University is committed to observing federal policies and regulations and Association for Assessment and Accreditation of Laboratory Animal Care (AAALAC) International standards and guidelines for humane care and use of animals. Federal guidelines, the Animal Welfare Act (AWA) and The Guide were followed when carrying out experiments. Procedures involved euthanasia and harvesting of bone marrow macrophages. All efforts were made to minimize discomfort, pain and distress.

### Bacteria

Frozen stocks of *P. gingivalis* wild-type strain 381 and the lipid A mutants (*PG1587*_381_ and *PG1773*_381_) were grown anaerobically at 37°C on blood agar plates (Remel) for 3–5 days [73]. Brain heart infusion broth (Becton-Dickinson Biosciences) supplemented with yeast extract (0.5%; Becton-Dickinson Biosciences), hemin (10 µg/ml; Sigma-Aldrich), and menadione (1 mg/ml; Sigma- Aldrich) was inoculated with plate grown bacteria and cultures grown anaerobically for 16–18 h. *P. gingivalis* lipid A mutants were grown in the presence of erythromycin (5 µg/mL).

### Gene deletions in *P. gingivalis* strain 381

The genomic nucleotide sequences encoding the putative lipid A 1-phosphatase, *PG1773*, and the putative lipid A 4′-phosphatase, *PG1587*, were obtained from searches of the annotated *P. gingivalis* W83 genome at The Comprehensive Microbial Resource (http://cmr.jcvi.org/tigr-scripts/CMR/CmrHomePage.cgi). Gene deletions were created by introducing an erythromycin resistance cassette (*ermF/AM*) in place of the coding region for *PG1773* and *PG1587*. Polymerase chain reaction (PCR) amplification of genomic DNA from *P. gingivalis* 381 was performed using primer sets designed against the W83 sequence to amplify 1000 base-pairs upstream and 1000 base-pairs downstream from the regions adjacent to the *PG1773* and *PG1587* coding regions, respectively. The amplified 5′ and 3′ flanking regions for *PG1773* and *PG1587*, respectively, were co-ligated with the *ermF/AM* cassettes respectively into pcDNA3.1(−) to generate the gene disruption plasmids, p1773 5′flank:erm:3′flank and p1587 5′flank:erm:3′flank. *P. gingivalis* 381 deficient in either *PG1587* (*PG1587*_381_) or *PG1773* (*PG1773*_381_) was generated by introducing either p1587 5′flank:erm:3′flank or p1773 5′flank:erm:3′flank into *P. gingivalis* 381 by electroporation in a GenePulser Xcell (BioRad, Hercules, CA). Bacteria were plated on TYHK/agar plates containing the appropriate selective medium, which included erythromycin (5 µg/ml), and incubated anaerobically. One week later, colonies were selected for characterization. Loss of the *PG1587* and *PG1773* coding sequences were confirmed in all clones by PCR analyses using primers designed to detect the coding sequences in wild-type 381 bacteria.

### MALDI-TOF MS analyses

LPS and Lipid A from *P. gingivalis* 381 and the lipid A mutant strains (*PG1587*_381_ and *PG1773*_381_) were isolated as previously described [23]. For MALDI-TOF MS analyses, lipid A were analyzed in the negative ion mode on an AutoFlex Analyzer (Bruker Daltonics). Data were acquired and processed using Flex Analysis software (Bruker Daltonics) [6], [23].

### HEK293 cell TLR activation assays

HEK293 cells were plated in 96-well plates at a density of 4×10^4^ cells per well, and transfected the following day with plasmids bearing firefly luciferase, *Renilla* luciferase, recombinant murine TLR4 and MD-2 or recombinant murine TLR2 and TLR1 by standard calcium phosphate precipitation. After overnight transfection, the test wells were stimulated in triplicate for 4 hours at 37°C with the indicated doses of LPS isolates or live bacteria. Following stimulation, the transfected HEK293 cells were rinsed with phosphate-buffered saline and lysed with 50 µl of passive lysis buffer (Promega, Madison, WI). Luciferase activity was measured using the Dual Luciferase Assay Reporter System (Promega, Madison, WI). Data are expressed as fold increase of NF-κB-activity which represents the ratio of NF-κB-dependent fire-fly luciferase activity to β-Actin promoter-dependent *Renilla* luciferase activity.

### Polymyxin B sensitivity assays

BHI broth cultures of wild-type 381 and the lipid A mutant strains were started at an optical density of .1 at 660 nm in the presence or absence of increasing concentrations (1, 5, 10, 25, 50 µg/mL) of polymxin B (InvivoGen). After overnight growth under anaerobic conditions, growth was assessed spectrophotometrically at 660 nm [74].

### Animals

Male ApoE^−/−^ and C57BL/6 mice were obtained from The Jackson Laboratory (Bar Harbor, ME). C57BL/6 mice deficient in TLR2 and TLR4 were provided by Dr. S. Akira (Osaka University, Osaka, Japan) and bred in house. Mice were maintained under specific-pathogen free conditions and cared for in accordance with the Boston University Institutional Animal Care and Use Committee.

### Bone marrow derived macrophage culture and stimulation

Bone marrow derived macrophages (BMDM) from wild-type and knockout mice were cultured in RPMI with 10% fetal bovine serum (Thermo Scientific HyClone Fetal Bovine Serum (U.S.), Defined SH3007003HI Heat inactivated) and 20% L929 supernatants [49] and were allowed to mature into macrophages over 7 days. Cells were seeded into 24-well plates at 2×10^5^ cells/well (ELISA assays) or 6-well plates at 1×10^6^ cells/well (Western blot analysis) and stimulated with bacteria at indicated MOI overnight. Cells stimulated with LPS from *E. coli* OIII:B4 (InvivoGen) or Pam3CysSk4 (InvivoGen) served as controls.

### ELISA

Levels of IL-1β, TNFα, IL-6 (BD Bioscience), IL-1α (eBioscience) and KC (R&D Systems) in cell culture supernatants were analyzed by ELISA.

### Immunoblotting

Proteins from cell culture supernatants were precipitated with ethanol at −20°C overnight and resuspended in Laemmli sample buffer. Cellular lysates were collected in RIPA buffer (Thermo Scientific) and samples were prepared in Laemmli sample buffer. Samples were separated by SDS-PAGE, transferred to polyvinyldifluoride membranes, blocked in 5% milk and target proteins were detected using antibodies to IL-1β (H-153) and caspase-1 p10 (M-20) (Santa Cruz Biotechnology). An antibody to β-actin (A1978 Sigma) was used as the loading control.

### Antibiotic protection assay

BMDM were stimulated with *P. gingivalis* or lipid A mutants for 2 h and 6 h. Extracellular nonadherent bacteria were removed by washing with PBS. Adherent bacteria were killed by addition of gentamicin (300 µg/mL) and metronidazole (200 µg/mL) for 1 hr. After PBS wash, BMDM were lysed with HyClone water (Thermo Scientific) for 10 min. Serial dilutions of the lysate were plated on blood agar plates and cultured anaerobically for CFU enumeration [65].

### Oral challenge

Mice were fed a normal chow diet (Global 2018; Harlan Teklad, Madison, WI). Six-week old male mice were treated with a 10-day regimen of oral antibiotics to allow for *P. gingivalis* colonization. Mice were challenged by oral application of vehicle (2% carboxymethylcellulose in PBS) or the *P. gingivalis* strains (1×10^9^ CFU) at the buccal surface of the maxilla 5 times a week for 3 weeks [36], [37].

### *In vivo* mouse Magnetic Resonance Angiography (MRA) and data analysis

*In vivo* imaging of the innominate artery was performed using a vertical-bore Bruker 11.7 T Avance spectrometer (Bruker; Billerica, MA) as previously described [53]. Mice were anesthetized with 0.5–2% inhaled isoflurane and placed in a vertical 30 mm probe (Micro 2.5). Respiration was monitored using a small animal monitoring and gating system (SA Instruments, Waukesha, WI). The angiography data was acquired with a fast low-angle shot (FLASH) sequence using the following parameters: slab thickness = 1.5 cm; flip angle = 45°; repetition time = 20 ms; echo time = 2.2 ms; field of view = 1.5×1.5×1.5 cm; matrix = 128×128×128; number of average = 4. The total scan time was ∼25 min. Visualization of the vasculature was achieved by 3D maximum intensity projections (MIP) of angiographic images reconstructed using Paravision. The target cross section of the innominate artery was chosen at 0.3- to 0.5-mm distance below the subclavian bifurcation. Lumen area of the chosen cross section was manually defined and calculated with ImageJ (National Institutes of Health) by two independent observers. The intra-reader reliability was excellent with interclass correlation coefficient values of 0.91.

### Immunohistochemistry

Mice were euthanized (n = 3–4/group), perfused with PBS (5 mL) and the aortic arch with heart tissue was embedded in OCT freezing compound. Seven-micrometer serial cryosections were collected every 70 µm in the innominate artery. Immunohistochemistry was performed on cryosections corresponding to greatest plaque accumulation in the innominate artery as previously described [42], [53] using rat anti-mouse F4/80 (no. MCA497R; Serotec, Oxford, U.K.) or isotype controls (no. MCA1125; Serotec). Biotinylated anti-rat (mouse absorbed) IgG was used as secondary Ab (Vector Laboratories, Burlin- game, CA). Nuclei were counter-stained with hematoxylin. Digital micrographs were captured at 10× and 40×.

### Atherosclerotic plaque assessment

Aortas were harvested and stained with Oil Red O as described [42]. Digital micrographs were taken, and total area of atherosclerotic plaque was determined using ImageJ (NIH) by a blinded observer.

### Microcomputed tomography

Three-dimensional analysis of alveolar bone loss was assessed as previously described [38]. Briefly, cephalons were fixed for 24–48 h in 4% buffered paraformaldehyde and stored at 4°C in 70% ethanol until evaluation by microcomputed tomography (micro-CT). Quantitative three-dimensional analysis of alveolar bone loss in hemi-maxillae was performed using a desktop micro-CT system (μCT 40; Scanco Medical AG, Bassersdorf, Switzerland). Maxillary block biopsies were scanned at a resolution of 12 µm in all three spatial dimensions. Raw images were converted into high-quality dicoms and analyzed using computer software (Amira 5.2.2; Visage Imaging). Residual supporting bone volume was determined for the buccal roots. The apical basis of the measured volume was set mesio-distally parallel to the cemento-enamel junction and bucco-palatinally parallel to the occlusal plane. Results represent residual bone volume (mm^3^) above the reference plane (180 µm from the cemento-enamel junction).

### Statistical analysis

Data were analyzed by two-tailed unpaired Student's t test or ANOVA with Bonferroni's posttest where indicated. A p value of .05 was considered indicative of statistical significance.

## Supporting Information

Figure S1 **Fimbriae expression, gingipain activity, and growth of** ***P. gingivalis*** **lipid A 1- and 4′ phosphatase mutants.** Electron microscopy was performed with *P. gingivalis* wild-type strain 381, *PG1587*_381_ and *PG1773*_381_ (**A**). Fimbriae expression was examined by Western blot analysis in whole cell lysates (5×10^7^ CFU) using a monoclonal Ab to major fimbriae (**B**) [73]. The proteolytic activities of the cell-associated cysteine proteases, gingipain R (**RGP**) and gingipain K (**KGP**) for wild-type 381 and the lipid A mutants *PG1587*_381_ and *PG1773*_381_ in whole cultures (lysate) and supernatant fractions were determined by an *in vitro* gingipain assay [75] (**C**). Percent proteolytic activity as compared to *P. gingivalis* wild-type strain 381 gingipain activity for *PG1587*_381_ (**dotted**) and *PG1773*_381_ (**dashed**). Bars indicate mean ± SEM from three independent experiments. Brain heart infusion broth cultures of *P. gingivalis* wild-type (**solid**), *PG1587*_381_ (**dotted**), and *PG1773*_381_ (**dashed**) were inoculated at a starting OD of 0.3. Growth was monitored at indicated time points over 48 h (n = 4) (**D**).
(TIF)

Figure S2 **TLR2 and TLR4 contribute to the production of IL-1β and IL-1α in macrophages stimulated with** ***P. gingivalis*** **wild-type strain 381 and the lipid A mutant strains.** BMDMs from wild-type C57BL/6 (**white**), TLR2-deficient (**gray**), or TLR4-deficient mice (**black**) were stimulated with *P. gingivalis* wild-type strain 381 or the lipid A mutant strains *PG1587*_381_ and *PG1773*_381_ at an MOI of 100 and levels of IL-1β (**A**) and IL-1α (**B**) were assessed by ELISA. Bars indicate mean ± SEM for n = 3 sample wells. *p<.05 **p<.01; two-tailed unpaired t-tests.
(TIF)

Figure S3 **Plaque accumulation in the innominate artery following oral infection of ApoE^−/−^ mice with** ***P. gingivalis*** **.** Representative images of the innominate artery with hematoxylin and eosin staining for each group at 10× and 40× (n = 3/group).
(TIF)

Figure S4 **Humoral response following oral infection of ApoE^−/−^ mice with** ***P. gingivalis.*** *P. gingivalis*-specific Ab isotypes IgG1 (**A**), IgG2b (**B**), IgG2c (**C**) and IgG3 (**D**) were measured in serum by ELISA at 16 wk post-infection of ApoE^−/−^ mice with *P. gingivalis* wild-type strain 381 and the lipid A mutants *PG1587*_381_ and *PG1773*_381_ (n = 10–12 mice/group). * p<.05 **p≤.001; two-tailed unpaired t-tests.
(TIF)

Text S1 **Supporting information on experimental procedures.** Describes the experimental procedures utilized for the supplemental data. This includes experimental procedures for Electron Microscopy, Gingipain Assay, ELISA, and Histology.
(DOCX)
