# Supplementary figures and images for: Distinct Lipid A Moieties Contribute to Pathogen-Induced Site-Specific Vascular Inflammation
Source: PLoS Pathog. 2014 Jul 10;10(7):e1004215. doi: 10.1371/journal.ppat.1004215 (PMC4092147; doi:10.1371/journal.ppat.1004215)

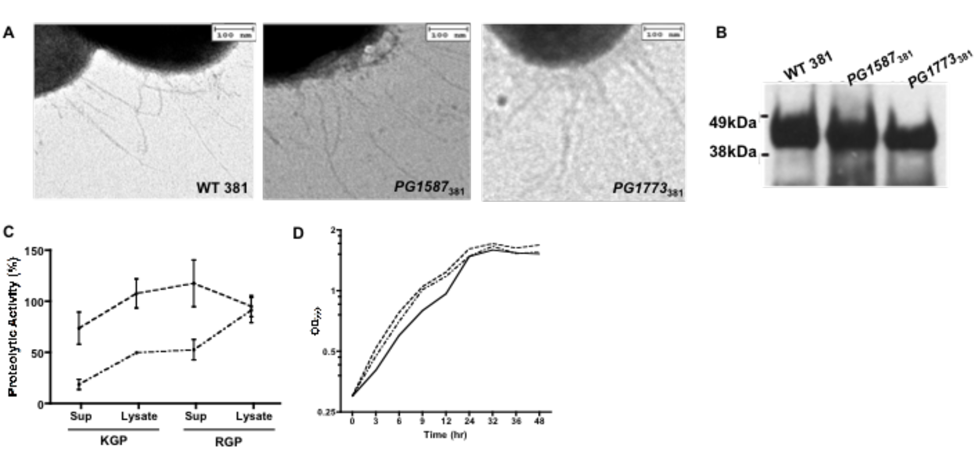

Supplement: Figure S1 — Fimbriae expression, gingipain activity, and growth of P. gingivalis lipid A 1- and 4′ phosphatase mutants. Electron microscopy was performed with P. gingivalis wild-type strain 381, PG1587 381 and PG1773 381 (A). Fimbriae expression was examined by Western blot analysis in whole cell lysates (5×10^7^ CFU) using a monoclonal Ab to major fimbriae (B) [73]. The proteolytic activities of the cell-associated cysteine proteases, gingipain R (RGP) and gingipain K (KGP) for wild-type 381 and the lipid A mutants PG1587 381 and PG1773 381 in whole cultures (lysate) and supernatant fractions were determined by an in vitro gingipain assay [75] (C). Percent proteolytic activity as compared to P. gingivalis wild-type strain 381 gingipain activity for PG1587 381 (dotted) and PG1773 381 (dashed). Bars indicate mean ± SEM from three independent experiments. Brain heart infusion broth cultures of P. gingivalis wild-type (solid), PG1587 381 (dotted), and PG1773 381 (dashed) were inoculated at a starting OD of 0.3. Growth was monitored at indicated time points over 48 h (n = 4) (D). (TIF) [file ppat.1004215.s001.tif]

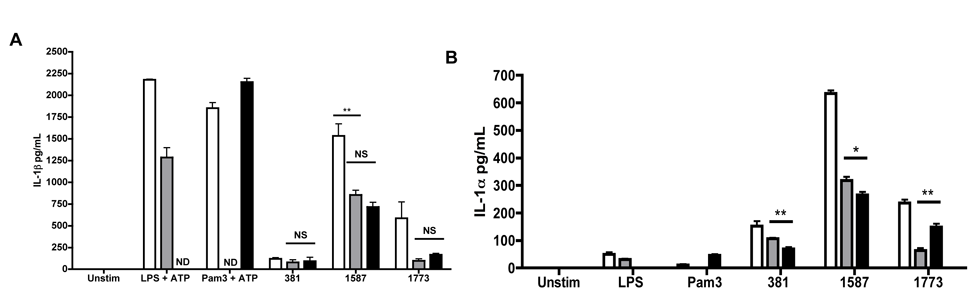

Supplement: Figure S2 — TLR2 and TLR4 contribute to the production of IL-1β and IL-1α in macrophages stimulated with P. gingivalis wild-type strain 381 and the lipid A mutant strains. BMDMs from wild-type C57BL/6 (white), TLR2-deficient (gray), or TLR4-deficient mice (black) were stimulated with P. gingivalis wild-type strain 381 or the lipid A mutant strains PG1587 381 and PG1773 381 at an MOI of 100 and levels of IL-1β (A) and IL-1α (B) were assessed by ELISA. Bars indicate mean ± SEM for n = 3 sample wells. *p<.05 **p<.01; two-tailed unpaired t-tests. (TIF) [file ppat.1004215.s002.tif]

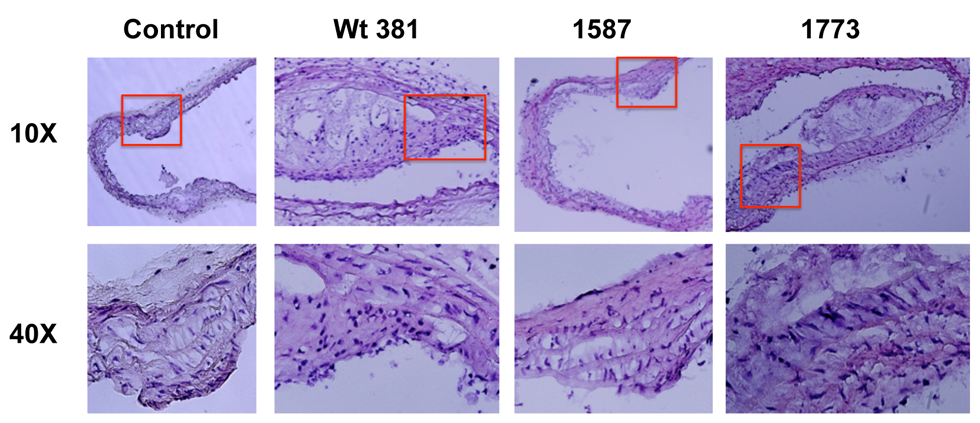

Supplement: Figure S3 — Plaque accumulation in the innominate artery following oral infection of ApoE−/− mice with P. gingivalis . Representative images of the innominate artery with hematoxylin and eosin staining for each group at 10× and 40× (n = 3/group). (TIF) [file ppat.1004215.s003.tif]

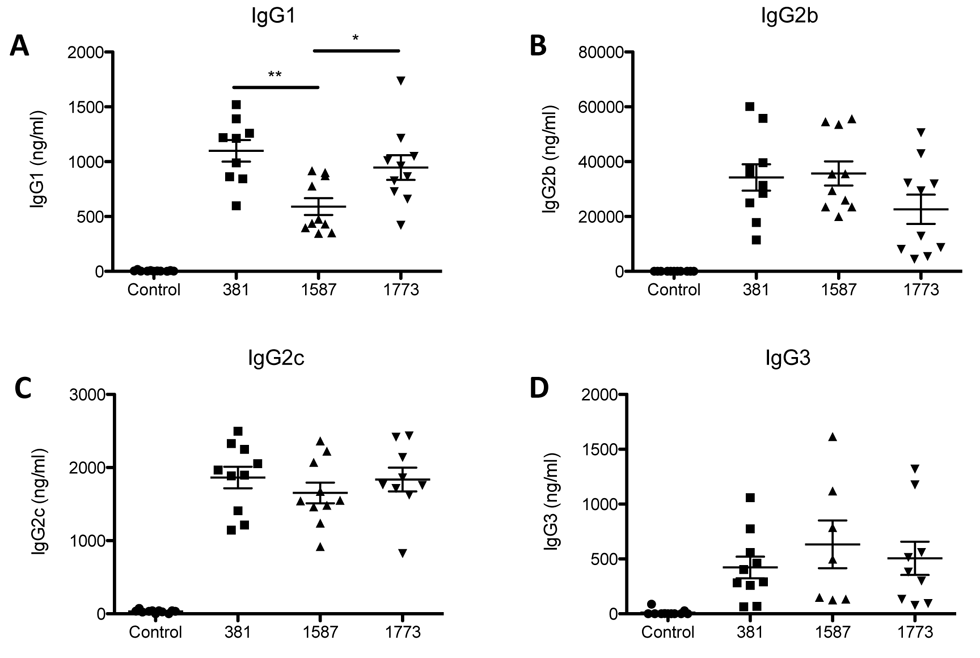

Supplement: Figure S4 — Humoral response following oral infection of ApoE−/− mice with P. gingivalis. P. gingivalis-specific Ab isotypes IgG1 (A), IgG2b (B), IgG2c (C) and IgG3 (D) were measured in serum by ELISA at 16 wk post-infection of ApoE−/− mice with P. gingivalis wild-type strain 381 and the lipid A mutants PG1587 381 and PG1773 381 (n = 10–12 mice/group). * p<.05 **p≤.001; two-tailed unpaired t-tests. (TIF) [file ppat.1004215.s004.tif]
