# Supplementary material for: Distinct Lipid A Moieties Contribute to Pathogen-Induced Site-Specific Vascular Inflammation
Source: PLoS Pathog. 2014 Jul 10;10(7):e1004215. doi: 10.1371/journal.ppat.1004215 (PMC4092147; doi:10.1371/journal.ppat.1004215)
Supplement: Text S1 — Supporting information on experimental procedures. Describes the experimental procedures utilized for the supplemental data. This includes experimental procedures for Electron Microscopy, Gingipain Assay, ELISA, and Histology. (DOCX) [file ppat.1004215.s005.docx]

**Experimental Procedures**

**Electron Microscopy**

Samples of *P. gingivalis* 381 and lipid A mutants were prepared by negative staining with phosphotungstic acid (neutral pH), and fimbriae were visualized by transmission electron microscopy.

**Gingipain Assay**

Lysine-specific (KGP) and arginine-specific (RGP) proteolytic activity of *P. gingivalis* 381 and lipid A mutant strains was determined in whole cultures and supernatant fractions with either N-benzoyl-L-arginine-p-nitroanilide or Z-lysine-p-nitroanilide. Kinetics of p-nitroanilide formation were measured spectrophotometrically at 405nm for 20 min and the Vmax was calculated in the linear range of the curve in order to assess change in mOD per min.

**ELISA**

Levels of IL-1β (BD Bioscience) and IL-1α (eBioscience) in cell culture supernatants were analyzed by ELISA. Furthermore, ELISA was utilized to assay *P. gingivalis*-specific Ab isotypes IgG1, IgG2b, IgG2c and IgG3 as previously described [1,2]. Briefly, *P. gingivalis* 381 and the lipid A mutants were fixed in 4% paraformaldehyde. Immulon 4HXB plates were coated with 10μg/mL of fixed bacteria. Serial dilutions of mouse serum collected at 16wks post infection were plated. Determination of IgG isotypes was performed using the C57BL/6 Clonotyping Kit (SouthernBiotech).

**Histology**

Mice were euthanized (n = 3-4/group), perfused with PBS (5mL) and the aortic arch with heart tissue was embedded in OCT freezing compound. Seven-micrometer serial cryosections were collected every 70μm in the innominate artery. Hematoxylin and eosin staining was performed on cryosectections corresponding to greatest plaque accumulation in the innominate artery as previously described [2,3]**.** Digital micrographs were captured at 10X and 40X.

**References**

1. Gibson FC, Hong C, Chou H-H, Yumoto H, Chen J, et al. (2004) Innate immune recognition of invasive bacteria accelerates atherosclerosis in apolipoprotein E-deficient mice. Circulation 109: 2801–2806. doi:10.1161/01.CIR.0000129769.17895.F0.

2. Hayashi C, Papadopoulos G, Gudino CV, Weinberg EO, Barth KR, et al. (2012) Protective Role for TLR4 Signaling in Atherosclerosis Progression as Revealed by Infection with a Common Oral Pathogen. The Journal of Immunology 189: 3681–3688. doi:10.4049/jimmunol.1201541.

3. Hayashi C, Viereck J, Hua N, Phinikaridou A, Madrigal AG, et al. (2011) Porphyromonas gingivalis accelerates inflammatory atherosclerosis in the innominate artery of ApoE deficient mice. Atherosclerosis 215: 52–59. doi:10.1016/j.atherosclerosis.2010.12.009.
